# Assessing the effects of artificial gravity in an analog of long-duration spaceflight: The protocol and implementation of the AGBRESA bed rest study

**DOI:** 10.3389/fphys.2022.976926

**Published:** 2022-09-08

**Authors:** Gilles Clément, Jörn Rittweger, Andrea Nitsche, Wolfgang Doering, Petra Frings-Meuthen, Olga Hand, Timo Frett, Alexandra Noppe, Freia Paulke, Leopold Lecheler, Jens Jordan, Claudia Stern, Edwin Mulder

**Affiliations:** ^1^ KBR, Houston, TX, United States; ^2^ Institute of Aerospace Medicine, German Aerospace Center, Cologne, Germany

**Keywords:** bed rest, artificial gravity, weightlessness, countermeasures, analogs

## Abstract

A comprehensive strategy is required to mitigate risks to astronauts’ health, well-being, and performance. This strategy includes developing countermeasures to prevent or reduce adverse responses to the stressors astronauts encounter during spaceflight, such as weightlessness. Because artificial gravity (AG) by centrifugation simultaneously affects all physiological systems, AG could mitigate the effects of weightlessness in multiple systems. In 2019, NASA and the German Aerospace Center conducted a 60-days Artificial Gravity Bed Rest Study with the European Space Agency (AGBRESA). The objectives of this study were to 1) determine if 30 min of AG daily is protective during head down bed rest, and 2) compare the protective effects of a single daily bout (30 min) of AG versus multiple daily bouts (6 × 5 min) of AG (1 Gz at the center of mass) on physiological functions that are affected by weightlessness and by head-down tilt bed rest. The AGBRESA study involved a comprehensive suite of standard and innovative technologies to characterize changes in a broad spectrum of physiological systems. The current article is intended to provide a detailed overview of the methods used during AGBRESA.

## Introduction

Before humans embark on a new era of deep space exploration and missions to Mars, efficient measures must be developed to promote the health and the performance of these explorers. In weightlessness, the human body is deprived of static gravitational acceleration along the longitudinal body axis (Gz), and this gravitational unloading results in headward fluid shift (Vernikos et al., 1996), decreased muscular capacity (Edgerton and Roy, 1994), loss of bone density (Leblanc et al., 2007), orthostatic intolerance (Meck et al., 2001), and impaired postural and locomotor control (Cohen et al., 2012). Head-down tilt (HDT) bed rest mimics the cephalad fluid shift and body unloading that occurs during spaceflight, and therefore, bed rest has been widely used to investigate the mechanisms underlying weightlessness-associated changes in humans, and to test countermeasures for alleviating the deleterious physiological adaptations to weightlessness (Hargens and Vico, 2016).

Artificial gravity (AG) by centrifugation has long been considered a potential countermeasure to combat the adverse effects of weightlessness. AG is regarded as a countermeasure that addresses multiple integrated systems affected by Gz unloading (Clément and Pavy-le Train, 2004). Specifically, AG evokes cardiovascular responses and provides mechanical loading on bone and on antigravity muscles. AG also stimulates proprioception and the otoliths of the vestibular system. Daily AG exposure has been shown to mitigate orthostatic intolerance and maintain exercise capacity after 5–30 days of HDT bed rest (Yajima et al., 1994; Iwasaki et al., 1998; Iwase, 2005; Linnarsson et al., 2015; see Clément et al., 2016 for a review). The Artificial Gravity Bed Rest Study with the European Space Agency (AGBRESA) was designed to evaluate the efficacy of AG to mitigate physiological deconditioning during 60 days of bed rest.

The AG interventions in the AGBRESA study consisted of a 30-min daily exposure to centripetal acceleration during short-radius centrifugation with the subjects in the supine position. This centrifugation generated 1 Gz at the body’s center of mass (CoM) and approximately 2 Gz at the feet. This acceleration induces considerable blood pooling toward the feet, cardiovascular homeostasis (Stenger et al., 2012), and potentially pain, which could affect the subject’s tolerance of AG over an extended period. For this reason, we opted to investigate the *minimum* daily duration of AG that can effectively mitigate deleterious physiological adaptations during 60 days of bed rest. Based on the previous ESA bed rest and artificial gravity study (BRAG-1) which used 30 min of AG daily (Linnarsson et al., 2015), the AGBRESA study included two different interventions to deliver a 30-min daily dose of AG: 1) continuously in one single bout (cAG); or 2) intermittently in six bouts of 5 min (iAG). The rationale for iAG was based on the observations that interval training and intermittent high-load exercise are the most efficient methods to support adaptation to altered gravitational loading (Herbert et al., 1988; Vernikos et al., 1996).

The primary goal of AGBRESA was to compare the efficacy and tolerability of the cAG and iAG interventions for mitigating the adverse physiological effects induced by HDT bed rest as an analog of weightlessness. The secondary aim of AGBRESA was to document the subject’s subjective rating of comfort/discomfort, perceived exhaustion, perceived benefits, and any other psychological issues associated with the AG protocols.

AGBRESA was conducted as an international collaborative effort between the National Aeronautics and Space Administration (NASA), the European Space Agency (ESA), and the German Aerospace Center (DLR). More than 20 research proposals in the fields of cardiovascular, immunology, genetics, muscle and bone, ocular, vestibular, psychology, sleep, cognition, and behavioral performance were integrated into a comprehensive assessment of the long-term responses to AG and bed rest. Details on these studies are provided in Supplementary Material. The results of these studies will be published in separate articles. Two batteries of measures were also administered throughout the study: one determined by an International Working Group and referred to as the *International Standard Measures* (Sundblad and Orlov, 2014; Sundblad et al., 2016; Clément et al., 2022), and the other determined by ESA and called *Bedrest Core Data*. These standard measures include some of the tests routinely conducted on astronauts to evaluate their adaptive changes in physiological, psychological, and biological responses during spaceflight.

In this paper, we provide a detailed description of the study design, participant selection, bed rest conditions, countermeasures, health indices, and adverse events during the AGBRESA study. We also review the assessments of subjects’ susceptibility to motion sickness and their acceptability and compliance with the continuous and intermittent AG interventions. This information is useful for interpreting the results of the investigator studies and the standard measures conducted during the AGBRESA study.

## Materials and methods

### study design

The AGBRESA study was conducted in: envihab, a medical research facility at the Institute of Aerospace Medicine at DLR in Cologne, Germany. The study was carried out in 2 separate campaigns between March 2019 and December 2020. Each campaign lasted 88 days and comprised three experimental phases: 1) a 14-days baseline data collection (BDC) phase (BDC-14 through BDC-1); 2) 60 days of 6° HDT bed rest phase (HDT1 through HDT60); and 3) a 14-days recovery (R+) phase (R+0 through R+13). Twelve participants were enrolled in each campaign, meeting the maximum occupancy of the bed rest facility of:envihab. Additional ESA and DLR-supported science and medical measures were performed during four ambulatory follow-up sessions on R+28, R+90, R+360, and R+720.

**FIGURE 1:**
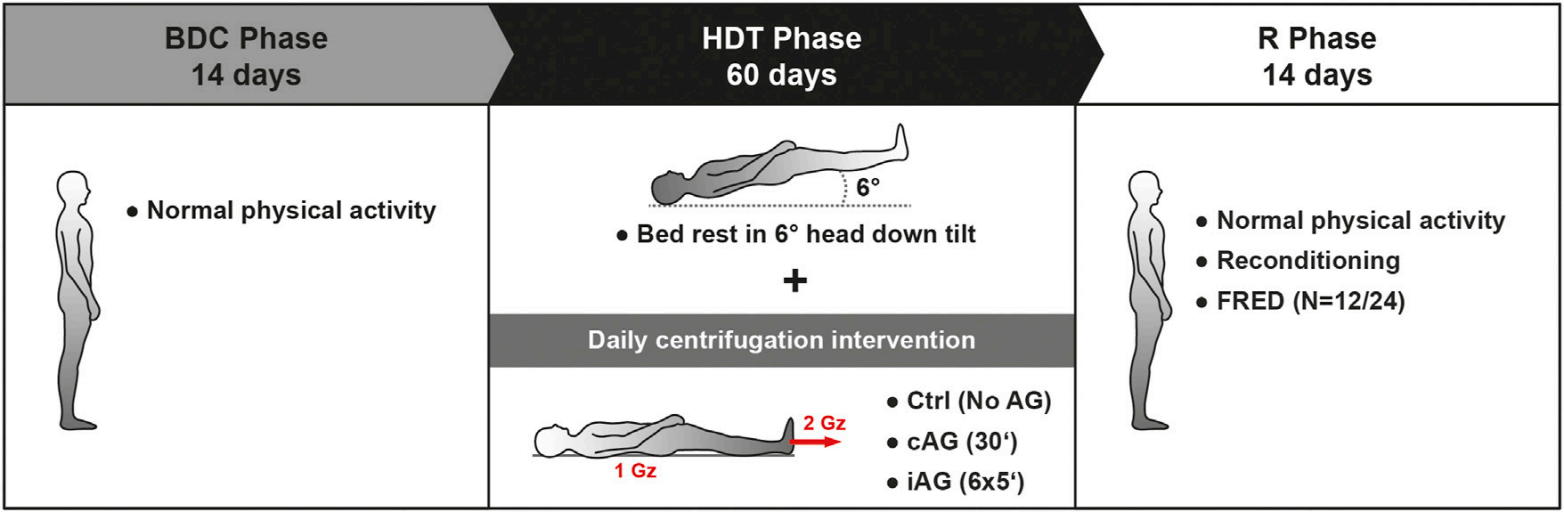
Schematic representation of the AGBRESA study design. FRED is a functional re-adaptive exercise device (Debuse et al., 2013) used during the recovery phase. Ctrl: control group, no centrifugation; cAG: continuous centrifugation for 30 min; iAG intermittent centrifugation for 6 × 5 min.

The participants were allocated to one of three experimental groups: 1) a passive control (Ctrl) group not exposed to centrifugation (*n* = 8); 2) a cAG group exposed to 30-min of continuous centrifugation (*n* = 8); and 3) an iAG group exposed to 6 × 5 min of centrifugation (*n* = 8). During the HDT phase, the 16 participants in the cAG and iAG groups were submitted to daily 30 min of supine centrifugation with a centripetal acceleration of 1 Gz at their CoM and approximately 2 Gz at their feet. Half of the subjects (*n* = 4) in each of the three groups, participated in daily training using a functional re-adaptive exercise device (Debuse et al., 2013) during the recovery phase (Figure 1).

Studies from ESA, NASA, and DLR were approved by their local institutional review boards. In addition, a protocol reflecting the integrated experiments was approved by the ethics commission of the local medical association (number 2018143, Ärztekammer Nordrhein) in Duesseldorf, Germany, as well as the Federal Office for Radiation Protection (number Z 5-22464/2018-074-R-G, Bundesamt für Strahlenschutz). The study was prospectively registered at the German Clinical Trials Register under number DRKS00015677.

### Participants

Selection inclusion criteria for the participants included: 1) physically and psychologically healthy men and women aged between 24 and 55 years old; 2) height within 153–190 cm; 3) body mass index (BMI) between 19–30 kg/m^2^; 4) non-smokers, or abstinence from smoking for at least 6 months prior to the start of the study; 5) possession of medical insurance; and 6) no criminal record. In addition, the candidates were required to confirm their availability for the duration of the study, including the follow-up examinations up to 2 years after bed rest, and their willingness to be assigned randomly either one of the interventions or to the control group.

Exclusion criteria included: 1) practice of strict vegetarian and vegan diet; 2) requirements for prescription medications, including contraceptives; 3) substance abuse; and 4) health conditions that would preclude participation, such as existing or history of cardiovascular dysfunction; musculoskeletal, ophthalmological, neurological, or psychiatric conditions; metabolic or endocrine disturbances (e.g., diabetes mellitus); blood clotting; pulmonary, sleep or pain disorders; gastroesophageal reflux; renal stones; or infectious or inflammatory diseases. Additionally, the candidates had to be free of any metallic implants that would interfere with MRI testing, and woman were required to have a normal length menstrual cycle (26–32 days).

**FIGURE 2:**
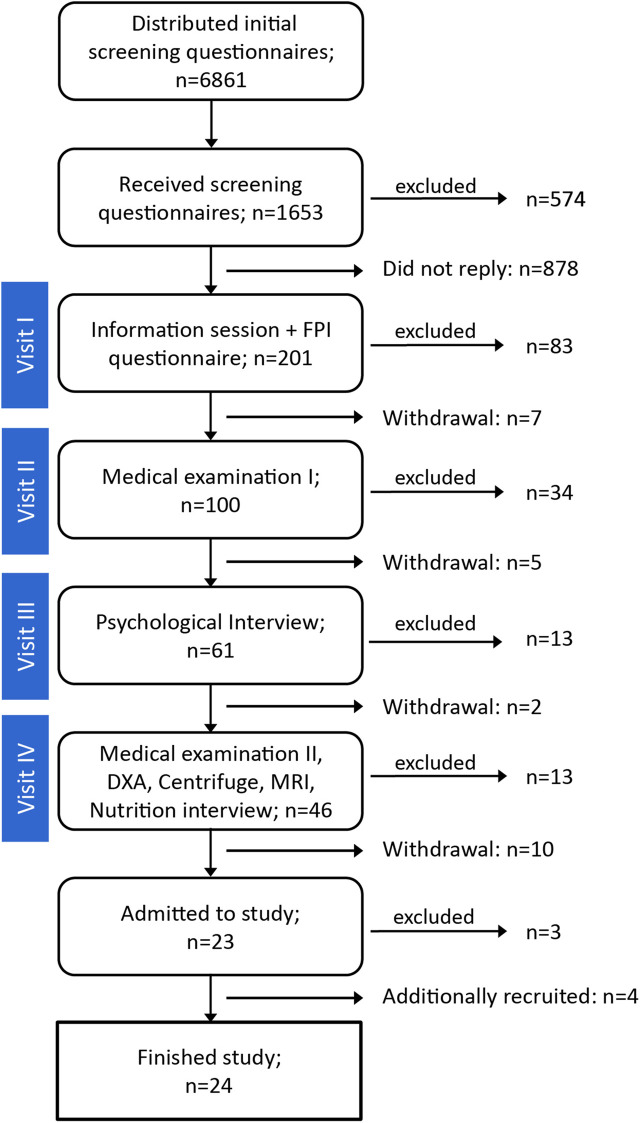
Number of candidates during the various steps of the selection process.

The subject recruitment process commenced once the study protocol had been approved by the local ethics committee. The call for AGBRESA participants was announced *via* media advertisements and social media. Eligible subjects in the DLR test subject registry were also contacted. The recruitment and screening process is outlined in Figure 2. The first step was to send an initial screening questionnaire to individuals who expressed interest in the study. Potential candidates meeting the minimum qualifying criteria were provided with study details, and thereafter, invited to attend a mandatory information session (Visit I) held at two different DLR locations in Cologne and in Hamburg. At the end of the information session, a preliminary psychological evaluation, the Freiburger Persönlichkeitsinventar (FPI) personality checklist (Fahrenberg et al., 2021), was collected from those who were willing to participate. If deemed suitable after the questionnaire assessments, candidates were invited to subsequent rigorous screening procedures including a modified Air Force Class III physical, psychological evaluation (Visit III), and a criminal background check. DLR physicians and psychologists who were experienced in bed rest subject selection conducted the examination.

The medical screening was conducted over two visits. During the first medical examination (Visit II), an extensive medical history was taken and fasting blood and urine samples were obtained for biochemical and drug tests and to screen for thyroid gland and kidney disorders. Additionally, resting and stress electrocardiogram (ECG), orthostatic testing, eye examinations, and pre-test genetic counseling for thrombophilia were conducted. Candidates who were medically eligible for the study subsequently underwent detailed psychological screening that involved questionnaires and interviews. In addition to the FPI, the Temperament Structure Scale (Maschke, 1987) and the 60-item NEO-FFI personality inventory (Costa and McCrae, 1992) were evaluated, along with the subject’s self-reported biography.

The second medical examination (Visit IV) consisted of a blood test for HIV, hepatitis, tuberculosis, and thrombophilia. The candidates were also screened for their ability to withstand the AGBRESA centrifugation protocol. For the first campaign, the candidates were exposed to 30 min of centrifugation with one Gz at the CoM and 1.4–1.6 Gz (depending on their height) at feet level. The spin-up acceleration was with 3.81 deg/s^2^, i.e., half that used during HDT (7.62 deg/s^2^). This screening protocol was revised for the second campaign to match the spin-up acceleration used during HDT. In addition, in-depth interviews of the candidates’ eating habits were conducted, and potential MRI contraindications such as permanent tattoos, medical and dental implants, and claustrophobia were examined. The recruitment process concluded with a dual energy X-ray absorptiometry (DXA) screening of bone mineral density in the femur and lumbar vertebra.

A total of 24 participants (16 males and eight females) completed the study. The study participants provided written informed consent before inclusion in the study and received monetary compensation for their time and effort participating in the study. The demographic information of the participants is provided in Table 1. The second campaign initially started with 11 participants due to last-minute dropouts. At the start of the study, three additional individuals were prematurely discharged on medical grounds: the diagnoses were not related to the study, and none of these individuals exhibited clinical signs or symptoms during the screening process. As a result, four additional participants were recruited and enrolled in the study to construct a full cohort of 12 for the second campaign. These four new subjects started 3 weeks later than the other eight participants from the second campaign and completed the entire study protocol.

**TABLE 1:** Demographic information of the AGBRESA participants. All measures were recorded upon admission and are presented as Mean (SD). No statistically significant difference was detected for any of the group measures (*p* < 0.05). cAG: continuous AG; iAG: intermittent AG; Ctrl: Control group; BMI: body max index; m: males, f: females.

	Ctrl	cAG	iAG	Total
N	8 (6 m; 2 f)	8 (5 m; 3 f)	8 (5 m; 3 f)	24 (16 m; 8 f)
Age (years)	34.25 (7.85)	31.88 (9.75)	33.75 (10.78)	33.29 (9.17)
Height (cm)	177.04 (7.27)	172.50 (8.05)	174.10 (10.52)	174.55 (8.55)
Weight (kg)	79.40 (12.67)	71.80 (10.15)	71.40 (4.51)	74.20 (10.03)
BMI (kg/m^2^)	25.18 (2.58)	24.00 (1.71)	23.64 (1.61)	24.27 (2.04)

The participants were assigned in a semi-random fashion to one of the two intervention groups (cAG or iAG) or to the Ctrl group. The first campaign aimed to create groups randomly, balanced only by sex distribution, whereas the second campaign aimed to balance the age, sex, height, and weight distribution among the three experimental groups, while also complementing the demographics of the participants in the first campaign. The additional four subjects that were recruited during the ongoing second campaign were also allocated to a specific group to balance the intervention groups in terms of male/female distribution (5 m, 3 f), while also aiming to balance age and weight of participants between the groups. The group assignment was only revealed to the participants, staff, and scientists on the morning of HDT1, i.e., after all baseline data collection had been completed.

### Bed rest standard conditions

In accordance with the international guidelines for bed rest studies (Sundblad and Orlov, 2014), the experimental conditions were standardized to enable proper comparison of results among different bed rest studies and to enhance the utility of data sharing. Throughout the study, participants followed a standard day-night cycle: they were awakened at 06:30 a.m. and lights were turned off and network disconnected at 11:00 p.m. To provide a “Zeitgeber” supporting circadian rhythm regulation, the lighting in each subject’s room and in the residential areas were closely regulated. From 07:30 a.m. to 03:00 p.m., room lighting was maintained at approximately 270 lux (measured at the head of bed), which is comparable to the light in a dimly lit office. No napping was permitted during waking hours. The study schedule and operations followed Germany’s daylight savings time shift. The average temperature and humidity of the residential area were 22.8 ± 1.6°C and 49.5% ± 4.5%, respectively, for the first campaign, and 22.5 ± 1.6°C and 53.0% ± 13.3% for the second campaign.

General health indices such as blood pressure (BP), heart rate (HR) (Intellivue MMS X2), and body temperature (self-measured using standard digital thermometer) were measured every morning in fasting state immediately after waking. Body weight (Sartorius floor scale, CAPS four weighing platform, and Combics display) was assessed after the first morning void at around 07:00 a.m., and the pooled urine volume in the preceding 24 h up until that point was recorded. To obtain accurate and standardized body weights, the participants were weighed in the same clothes throughout all assessments and the weight of the clothes was subtracted afterwards. Daily body weight during the HDT phase was measured on a designated and calibrated 6° HDT gurney on the abovementioned floor scale. All standard general health indices were obtained between 06:30 a.m. and 07:30 a.m. During the baseline and recovery phases, the subjects were ambulatory; however, physical activity was restricted to free movement within the ward.

To ensure safety and well-being of the study participants, medical care was available 24 h a day. The medical staff conducted daily ward rounds to monitor the health status of the subjects, and all medications were monitored and recorded in the clinical report throughout the study. Careful attention was paid when administering medications to avoid potential confounding effects on testing. Parameters to assess safety were evaluated from blood and urine samples collected on BDC-14, HDT10, HDT30, HDT50, R+1, and R+10. Bi-weekly ocular, ear, nose, and throat examinations and audiometry were performed by medical specialists at the start and end of the HDT phase. Additionally, subjects participated in weekly individual consultation sessions with a psychologist.

All participants continuously maintained strict 6° HDT bed rest for 60 days. They were permitted to lie on their stomach, back, or side; however, they were instructed to keep their legs straight when lying on their backs. The participants were required to always keep at least one shoulder on the mattress. They were not permitted to raise, contract, or stretch their legs to minimize mechanical stimuli (other than during the AG sessions and physiotherapy). Participants were video-monitored around the clock to assure study compliance and safety.

Daily routine activities such as eating, washing, showering, using the toilet, and leisure activities were performed in the strict 6° HDT position. Participants were not permitted to prop up their heads when eating; the head was kept below heart level at all times. As a precautionary measure to prevent otitis externa, which is prevalent in bed rest studies, ear plugs were provided for showering and participants were advised to thoroughly dry their hair and adequately drain their ears after showering. Participants were also instructed to avoid any manipulation of the outer ear canal, for example inserting in-ear headphones.

**FIGURE 3:**
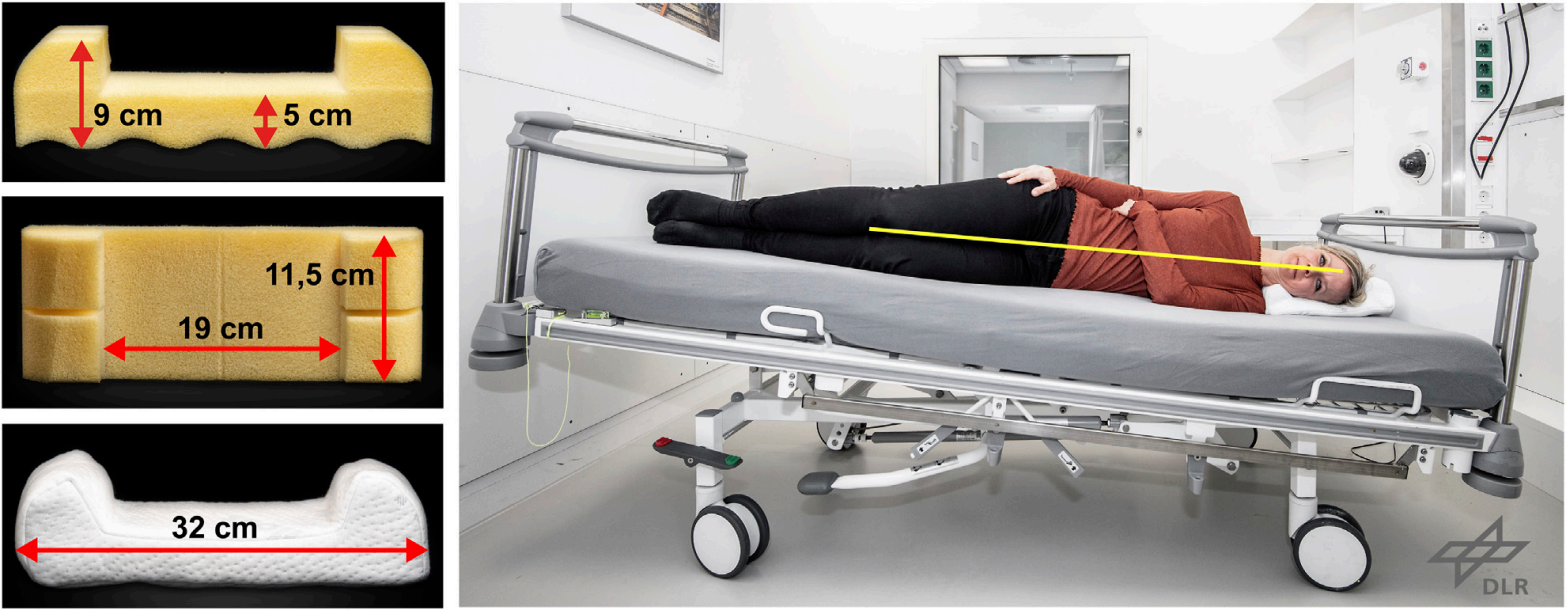
Details of the side pillow used to maintain the subjects’ head in the 6° HDT position when lying on their side.

To maintain the head in the 6° HDT position along the longitudinal axis during the HDT phase, the participants were not permitted to use a regular pillow. Only a dedicated side pillow was used when the subjects were lying on their side to keep the head from rolling along the *x*-axis (Figure 3). The side pillow was a modified Traumschloss Comfort neck support pillow (Gebers-Die Schlafexperten GmbH) made of cube cut visco foam (foam grade RG50). A customized pillowcase (double cloth 70% polyester, 30% viscose) contouring the shape of the foam was used for personal hygiene and to ensure the correct usage of the pillow. The correct usage of the side pillow was explained in detail to the subjects and its use was carefully monitored continuously through video surveillance cameras. The frequency of pillow usage at night was recorded and categorized (Table 2). The strict HDT bed rest and the use of only the side pillow were well tolerated by the participants over the 60 days of the study.

**TABLE 2:** Number of participants and pillow usage frequency for each of the study phases. Only the custom-made side pillow was allowed during HDT.

Pillow usage	BDC	HDT	R+
Always	19	8	16
Often	1	3	3
Sometimes	N/A	3	1
Occasional	1	4	N/A
Never	3	6	4
Total Count	24	24	24

On a few occasions during the HDT phase, subjects were placed in the horizontal position (0°) due to technical limits of the equipment or to obtain higher quality scientific data. This occurred during the cardiac and muscle/bone MRIs and during the bone mineral density measurements (DXA and tibial pQCT): each subject spent about 11 h in the horizontal position during HDT while these tests were being conducted. In addition, the daily centrifugation interventions (cAG and iAG) were performed in the horizontal position so the results could be compared with those of previous AG bed rest studies that also used centrifugation in supine (horizontal) position (Yajima et al., 1994; Iwasaki et al., 1998; Iwase, 2005; Linnarsson et al., 2015). Table 3 shows the total time each experimental group spent in the horizontal position during the HDT phase.

**TABLE 3:** Positional deviation from 6° HDT. The table indicates the number of hours during which the individuals in each experimental group were in the horizontal position (0°) during the 60-days HDT phase. These include the MRI, DXA, and pQCT exams, the AG interventions, and the rest periods between the AG runs during the iAG intervention.

		Ctrl	cAG	iAG
HDT1-HDT30	MRI, DXA, pQCT	4.5	4.5	4.5
	AG intervention		15	15
	Between AG runs			7.5
	Total	4.5	19.5	27.0
HDT31-HDT60	MRI, DXA, pQCT	6.5	6.5	6.5
	AG intervention		15	15
	Between AG runs			7.5
	Total	6.5	21.5	29.0
HDT1-HDT60	Total hours in horizontal position	11	41	56

### Centrifugation

All centrifugation sessions were performed on DLR’s 3.8-m radius short-arm human centrifuge (SAHC-1, AMST Systemtechnik, Austria Metall SystemTechnik). The participants lay on the centrifuge arm with their head towards the center of rotation and their feet pointing radially outwards. Body height ratios of 0.56 for males and 0.54 for females were used to determine the CoM of each subject (Gambino et al., 2006). The angular velocity of the centrifuge and the participant’s position relative to the center of rotation were individually adjusted to achieve 1 Gz at the CoM and approximately 2 Gz at the level of the feet. The rotation rate ranged from 29.1 rpm for the tallest subject to 32.2 rpm for the shortest subject. When assuming an ear-height ratio of 0.93, this rotation rate resulted in approximately 0.30 Gz at the inner ear level. The angular acceleration/deceleration of the centrifuge during spin-up and spin-down was 7.62 deg/s^2^, which required about 22 s for the centrifuge to reach constant velocity or to stop.

**FIGURE 4:**
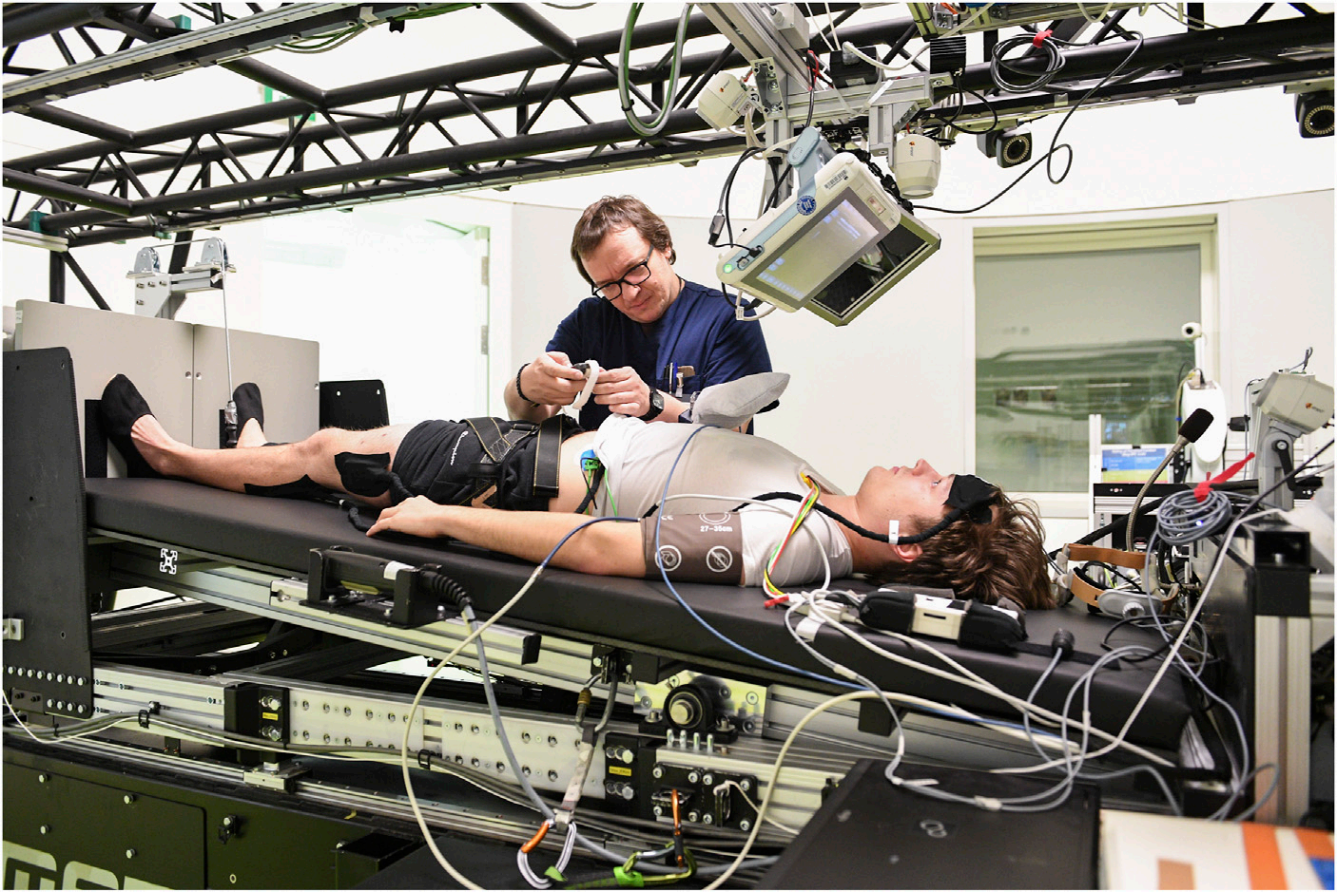
An operator is prepping a subject for a run on the DLR SAHC-1centrifuge. The subject is in HDT prior to centrifuge rotation.

The DLR SAHC-1 centrifuge included a 6° HDT nacelle to enable the subjects to be tilted before and after the centrifugation runs. Experiment preparations, including donning of equipment and safety checks, were performed while the subjects were in 6° HDT on the centrifuge (Figure 4). Participants were placed in the supine position (0°) just prior to the centrifugation runs. Immediately after the final stop of the centrifuge, the participants were hoisted to 6° HDT for doffing of equipment and recovery measurements. For the iAG intervention, the runs were separated by 3-min pauses (excluding spin-up and spin-down time), during which participants were kept in the supine position to avoid potential vestibular disturbances induced by frequent 6° HDT positional adjustments.

On BDC-11 and BDC-4, all 24 participants were familiarized with centrifugation 1 Gz at the CoM and 2 Gz at feet, over two 5-min runs separated by a 3-min break. Rotation was clockwise during the BDC-11 session, and counterclockwise during the BDC-4 session. During these familiarization sessions participants were trained to avoid making head movements and to fix their gaze on one point straight ahead. Additionally, to aid the participants in maintaining cardiovascular homeostasis, they were trained to recognize signs and symptoms of presyncope. They were instructed to contract their leg muscles regularly to avoid presyncopal symptoms associated with venous pooling.

The direction of centrifuge rotation alternated daily between clockwise and counterclockwise to prevent unbalanced loading between the left and right extremities over the course of the 60-days AG training. The centrifuge room was dimly lit during the centrifugation sessions. To promote engagement and relaxation during the centrifugation session, participants were permitted to listen to music or an audiobook of choice. However, no music was allowed during centrifugation sessions that combined experimental measurements.

All centrifugation runs were conducted between 09:00 a.m. and 07:00 p.m., and the time of the day of the AG runs (morning vs. afternoon) were counterbalanced for each subject. The participants wore a harness attached to the nacelle of the centrifuge to protect them from falling. Real-time measurements of the participant’s ECG, finger pulse oximetry, and HR, as well as BP (measured every 5 min using Philips IntelliVue MP2) were monitored by a medical doctor during all centrifuge runs. The participants’ face and leg movements were monitored by on-board cameras and two-way communication with the control room was available at all times. In the event of emesis, participants were instructed to turn their heads to the side and use the motion sickness bag placed next to their arm during rotations. The rotation was terminated if the subject requested to stop, if the subject was non-responsive, or had syncope, severe signs of orthostatic intolerance (self-reported nausea, sweating, dizziness, light-headedness), a sudden drop in systolic BP (>25 mmHg) or in HR (>15 bpm), irregular cardiac rhythm, or a low absolute systolic BP (<70 mmHg).

### Nutrition

All subjects consumed a strictly controlled diet for the entire duration of the study that was tailored to individual resting metabolic rates (RMRs) and body weight (BW). RMR was measured on BDC-14 by means of indirect calorimetry using the Weir Equation, and assuming 8.64 g/day 4) for urinary nitrogen (Metalyzer 3B, CORTEX Biophysik GmbH). Then, each participant’s total energy intake was set to 1.6 × RMR (corresponding to light intensity daily physical activities) during ambulatory phases and to 1.3 × RMR (corresponding to the energy need of a sedentary adult) during HDT. The total energy in the diet was adjusted by varying the fat and carbohydrate content to maintain the body weight within 3% of that measured on HDT3.

In line with international bed rest standards (Sundblad and Orlov, 2014), the energy intake for each participant was determined separately for protein, fat, carbohydrates as follows: 1) the energy intake of proteins was set at 1.2g/kgBW/d; 2) the energy intake of fat was set at 30–35% of the total energy intake; 3) the intake of fiber was set at a minimum of 30 g/d; and 4) the remaining energy intake was supplied through carbohydrates. Vitamins and minerals were also standardized and controlled. To account for the reduced sunlight exposure, 1,000 international units (IU)/d vitamin D were supplemented, in addition to the dietary vitamin D content.

Daily fluid intake from food and beverages was set at 50 ml/kgBW/d. Fluids were provided as water, juices, shakes, and caffeine-free and herbal-free tea. During the early recovery days (R+0 through R+5), water intake was increased due to an increased thirst caused by the shift in body fluid. The fluid intake was increased to 70 ml/kgBW/d for R+0 and R+1, and to 60 ml/kgBW/d for R+2 and R+3. From R+4 through R+11, the fluid intake was normalized back to 50 ml/kgBW/d but was augmented by 0.5 ml/kgBW/d and the daily energy intake was increased by 0.1 x RMR to account for the increased sweat and energy loss from the reconditioning sessions.

All meals were planned and supervised by nutritionists and dieticians and were prepared in the: envihab metabolic kitchen. Nutrient content of each prepared meal was calculated using PRODI software (Kluthe Prodi 6.10 Expert, Nutri-Science, Germany). Seven different menus for the ambulatory phase and 14 different menus for the HDT phase were planned, which consisted of standard German food items. The menus were standardized per study day and were served in three main meals and one snack throughout the day. The timing at which the meals and snacks were served was also in accordance with the food consumption restrictions specified for the AG sessions and experiments. Intake of caffeine, chocolate, black tea, alcohol, and herbal drinks, or other beverages not offered in the metabolic diet, were prohibited. Subjects were required to consume the entirety of the meals within 30 min and were instructed to consume only the diet provided by the project team.

The nutritional intakes during AGBRESA are listed in Table 4 along with the recommended intake of nutrients. In summary, the relative intake levels of protein, fat, and carbohydrates were approximately 15%, 32%, and 52%, respectively. The recommended values were achieved daily, with the exception of the vitamins and minerals, which were achieved weekly on average.

**TABLE 4:** Standardized nutritional intake for each experimental group (Mean ± SD). The AGBRESA nutritional requirements are presented in the right-most column for reference.

Nutrients	BDC	HDT	HDT	HDT	R	Nutritional req’ts
	All groups	Ctrl	cAG	iAG	All groups	
Total energy (kcal/d)	2,610 ± 379	2,431 ± 365	2,215 ± 362	2,266 ± 323	2,673 ± 417	Maintain body weight
Protein (g/d)	89.2 ± 11.4	93.56 ± 16.58	84.02 ± 13.54	85.69 ± 6.51	88.39 ± 13.19	—
Protein (g/kgBW/d)	1.2 ± 0.02	1.17 ± 0.1	1.18 ± 0.08	1.19 ± 0.05	1.19 ± 0.07	1.2 g/kgBW/d
Protein (% of total energy)	13.99 ± 1.34	15.61 ± 1.82	15.43 ± 1.27	15.52 ± 1.6	13.53 ± 1.5	—
Total fat (g/d)	90.61 ± 14.08	83.88 ± 14.26	75.77 ± 13.77	77.74 ± 12.32	92.97 ± 17	—
Total fat (% of total energy)	31.98 ± 0.97	31.56 ± 1.72	31.38 ± 1.46	31.49 ± 1.17	31.98 ± 1.92	30%–35% of total energy
Monosaturated fatty acids (g/d)	31.57 ± 6.53	29.06 ± 5.74	26.19 ± 5.76	26.99 ± 5.34	34 ± 8.82	—
Monosaturated fatty acids (% of total energy)	11.11 ± 1.25	10.95 ± 1.48	10.83 ± 1.28	10.92 ± 1.17	11.66 ± 1.85	≥10% of total energy
Saturated fatty acids (g/d)	24.16 ± 4.42	22.22 ± 4.99	20 ± 4.64	20.52 ± 4.23	24.38 ± 4.65	—
Saturated fatty acids (% of total energy)	8.55 ± 1.03	8.36 ± 1.38	8.29 ± 1.31	8.33 ± 1.23	8.43 ± 1.12	≤10% of total energy
Polyunsaturated fatty acids (g/d)	30.67 ± 6.41	28.59 ± 7.96	25.94 ± 7.03	26.5 ± 6.93	30.43 ± 6.89	—
Polyunsaturated fatty acids (% of total energy)	10.84 ± 1.66	10.74 ± 2.37	10.76 ± 2.31	10.75 ± 2.35	10.47 ± 1.61	≥7% of total energy
Carbohydrates (g/d)	329.2 ± 54.33	299.69 ± 46.03	273.71 ± 48	280.82 ± 48.13	339.94 ± 58.93	—
Carbohydrates (% of total energy)	51.16 ± 1.69	50.01 ± 2.25	50.09 ± 2	50.05 ± 2.01	51.59 ± 2.18	50%–60% of total energy
Total fiber (g/d)	37.13 ± 5.59	34.1 ± 5.86	33.98 ± 4.79	33.11 ± 4.65	38.57 ± 6.27	≥30 g/d
Fluid (ml/d)	3,741 ± 472	4,027 ± 488	3,557 ± 488	3,611 ± 228	4,358 ± 707	—
Fluid (ml/kgBW/d)	50.53 ± 2.02	51.04 ± 3.73	50.22 ± 1.21	50.2 ± 2.27	58.94 ± 7.11	50 ml/kgBW/d
Calcium (mg/d)	1,080 ± 22.97	1,080 ± 65.4	1,081 ± 39.4	1,081 ± 31.87	1,084 ± 30.52	1,000–1,200 mg/d
Chloride (mg/d)	4,951 ± 444	4,725 ± 473	4,678 ± 457	4,731 ± 357	4,972 ± 591	—
Sodium (mg/d)	2,950 ± 92	2,905 ± 203	2,885 ± 223	2,918 ± 152	2,952 ± 230	2,500–3,000 mg/d
Sodium (mmol/kgBW/d)	1.76 ± 0.26	1.63 ± 0.29	1.8 ± 0.27	1.77 ± 0.14	1.76 ± 0.28	—
Potassium (mg/d)	3,918 ± 552	3,715 ± 460	3,560 ± 478	3,586 ± 399	3,971 ± 565	3.0–5.0 g/d
Fluoride (mg/d)	2.16 ± 0.54	2 ± 0.75	2.11 ± 0.73	2.11 ± 0.71	2.12 ± 0.49	1.5–4 mg/d
Iodine (µg/d)	203.46 ± 73.24	208.55 ± 59.9	206.43 ± 55.44	208.75 ± 52.57	194.69 ± 68.56	≥200 μg/d
Copper (µg/d)	1915 ± 293	1904 ± 343	1807 ± 329	1835 ± 313	2023 ± 332	1,500–3,000 μg/d

### Other countermeasures

Self-imposed static and dynamic muscle contractions were not permitted; however, standards for bed rest studies stipulate that key muscle groups and joints must be stretched to avoid muscle contractures and to alleviate muscle stiffness and back pain.

All participants performed a 15-min stretching routine every morning. The routine included stretching of muscles surrounding the hip, knee, ankle, shoulder, wrist, and cervical lumbar spine, and isometric contractions of hip flexors and extensors, knee extensors, and abdominal muscles. Participants were trained to perform this body stretching routine on BDC-4 by a physiotherapist and were provided with resistance bands and graphic instructions.

Starting on HDT2, a 60-min afternoon physiotherapy session was also provided every other day during the HDT phase. Additional physiotherapy sessions were provided in the first week of the R+ phase, however, the sessions on R+2 through R+5 were reduced to 30 min to accommodate the data collection schedule. The physiotherapy was divided into 4 phases: 1) HDT1 to HDT20; 2) HDT21 to HDT40; 3) HDT41 to HDT60; and 4) and R+1 to R+5, and different areas of focus, pressure, and movements were prescribed by physiotherapists for each phase. Briefly, the first phase focused on back, neck, and shoulder discomfort, which typically occurs during the early stage of HDT. The second phase concentrated on the lower back to aid circulation of the lower extremities. The third phase focused on the feet, because foot discomfort due to disuse is often reported upon re-ambulation. Additional focus was aimed at circulation of the lower extremities to promote quick recovery. The fourth phase included rehabilitation prior to departure from the unit, and helped attenuate muscle discomfort the participants experience after resuming physical activity.

Various massage techniques to warm up and manipulate soft tissue, increase blood circulation, and joint mobilization were used for the standard regimen and to provide overall comfort to the subjects. In addition to the standard regimen, individual therapies were prescribed by the medical staff as needed when a medical issue was indicated.

Before re-ambulation, the participants were informed of the general procedures and safety measures to be practiced on R+0. The standardized re-ambulation procedure was built into the orthostatic tolerance test conducted as part of the International Standard Measures and Bedrest Core Data (Platts et al., 2009), and was performed on the DLR’s custom-built electronic tilt-table. After completing the orthostatic tolerance test, participants were monitored for an additional 15 min by medical staff. The participants were not permitted to stand or move their heads until the subsequent vestibular testing had been conducted. For safety purposes, and to limit the magnitude of delayed onset of muscle soreness, the participants were transported to various testing rooms in a wheelchair on R+0 and R+1. Participants were not permitted to shower on R+0, and they showered in the seated position on R+1.

During the recovery phase, the participants received physiotherapist-guided reconditioning training intended to gradually restore their range of motion, whole body strength, speed, coordination, core stability and body posture. Each participant received a daily 60-min rehabilitation session for 7 days between R+4 and R+11. The rehabilitation session started on R+4, after the main study measurements had been completed and when muscle soreness due to re-ambulation had subsided. R+8 was a rest day where no reconditioning was performed. Before the HDT phase, on BDC-4, all subjects were familiarized with the specifics of the reconditioning program, to help facilitate training during the weakened state after bed rest.

The rehabilitation sessions involved generic exercise regimen using a Bosu ball (Ashland, OH, United States) for balance and coordination training; disc cone hurdle and coordination ladder for plyometric agility drills; and a medicine ball and battle ropes for strength training. No rigorous physical interventions such as jogging and weight training were included in the protocol. The exercises started slowly and easily, progressed in speed and difficulty, and where tailored to each subject’s status. The rehabilitation program ended with dynamic stretches targeting the hamstrings, glutes, lower back, and the quadriceps muscle groups, and with physiotherapeutic exercises customized to the individual needs of the subject. During AGBRESA, four subjects (Ctrl: 1 f, 1 m; cAG: 1 f; iAG: 1 m) reported patellar tendon pain during the recovery phase. For these subjects, the reconditioning program was modified to avoid exercises that involved kneeling and jumping. Instead, their training focused on trunk stabilization that engaged the pain-free areas (e.g., abdominal and back exercises on the Bosu ball). These subjects also performed dynamic stretching and the coordination training on the ladder.

## Results

### Health indices and adverse events

The primary vital signs recorded before, during, and after bed rest were used to characterize the general health status of each subject. Measurements were obtained with the subjects at rest in the supine position (0°) during BDC and R+, and at 6° HDT during the HDT period. The vital signs were recorded first thing after waking up the subjects, in a standardized dimly lit environment, before the subjects participated in any experiments. These measurements were obtained several days after the participants acclimated to the transition between study phases. Time points were selected to coincide with standard measures and nutritional standardization. Data collected on HDT1, R+0, and R+13 (the day of discharge) were not used because the subjects’ psychological state could have affected their vital signs. Three distinct epochs were selected: 1) the BDC epoch included all measures obtained between BDC-6 and BDC-1; 2) the HDT epoch included all measures obtained between HDT3 and HDT60; and 3) the R+ epoch included all measurements obtained between R+6 and R+12. Analysis of variance (ANOVA) and linear mixed effect models were constructed to evaluate group differences in means and changes in health index over time. All analyses considered the effect of age and sex. The alpha level of significance for the analyses was set at 0.05. All statistical analyses were conducted using SPSS Statistic Version 21 (IBM SPSS Statistics for Windows v21.0, Armonk: NY, 2012).

**FIGURE 5:**
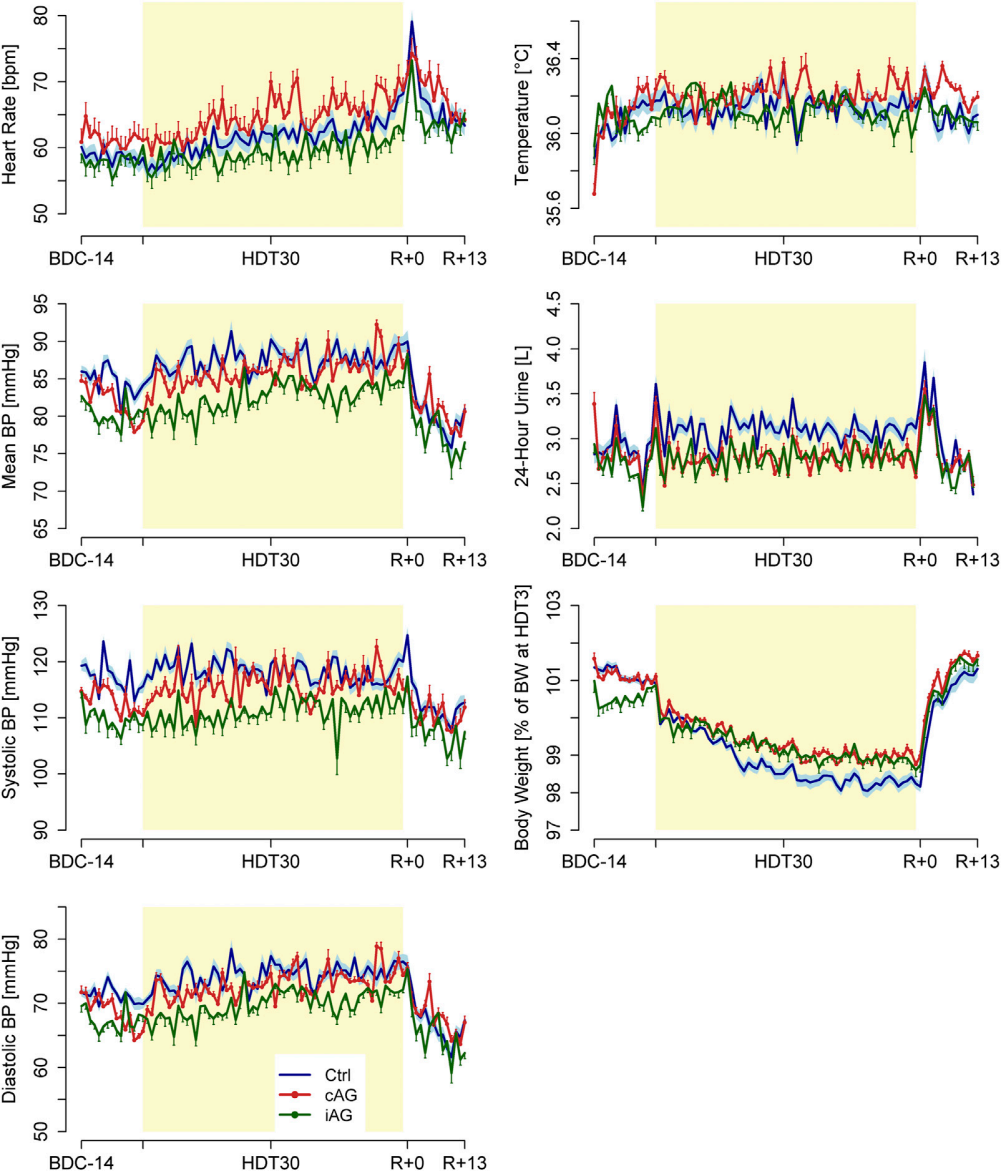
Daily group averages ± SD of the measured general health measurements for the three subject groups (8 subjects per group): heart rate, mean blood pressure, systolic blood pressure (BP), diastolic BP, temperature, 24-h urine volume, and body weight.

The daily group averages of the general health measurements are shown in Figure 5; Table 5 shows results of the general health measurements or each study group at baseline (BDC-1), at the start and end of the HDT phase (HDT3 and HDT60, respectively), and at the end of the recovery phase (R+12).

**TABLE 5:** Summary of the General Health Indices. Values were obtained through regression analysis and are presented for representative study dates as Mean ± SD.

		Baseline						Bed rest			Recovery		
	Group	BDC-1		*p*	HDT3		*p*	HDT60		*p*	R+12		*p*
Heart rate (bpm)	Ctrl	58.22	±8.54		58.25	±8.14		65.09	±8.63	†	64.39	±10.71	*
	cAG	61.40	±12.52	—	61.13	±11.36	—	68.31	±11.34	†	63.88	±10.09	—
	iAG	58.88	±9.67	—	57.40	±10.14	—	61.44	±6.45	—	62.45	±9.21	—
Mean arterial Pressure (mmHg)	Ctrl	84.08	±5.05		88.12	±6.42		90.02	±7.51	—	77.71	±8.50	*
	cAG	77.89	±3.41	—	84.18	±4.40	—	87.70	±3.09	—	77.67	±5.52	—
	iAG	80.72	±5.84	—	80.40	±4.44	—	84.76	±3.64	—	73.20	±4.62	*
Systolic blood pressure (mmHg)	Ctrl	114.98	±4.94		119.47	±6.08		117.34	±6.97	—	110.71	±7.71	—
	cAG	110.80	±9.02	—	114.77	±7.54	—	116.92	±6.76	—	108.57	±10.39	—
	iAG	109.80	±9.45	—	109.49	±6.95	a	112.93	±6.12	—	103.97	±8.81	*
Diastolic blood pressure (mmHg)	Ctrl	69.91	±5.10		73.33	±6.01		75.58	±6.96	—	63.61	±9.26	*
	cAG	64.43	±4.34	—	70.81	±5.11	—	74.99	±3.09	—	64.06	±5.82	—
	iAG	67.80	±5.06	—	67.60	±3.89	—	72.20	±3.47	—	61.00	±2.54	*
Temperature (°C)	Ctrl	36.19	±0.35		36.13	±0.29		36.15	±0.32	—	36.05	±0.42	—
	cAG	36.23	±0.31	—	36.19	±0.18	—	36.23	±0.16	—	36.14	±0.12	—
	iAG	36.03	±0.19	—	36.18	±0.29	—	36.08	±0.24	—	36.06	±0.19	—
Urine volume (ml)	Ctrl	2,801.54	±422.51		3,079.49	±505.81		3,103.47	±538.47	—	2,614.51	±335.41	—
	cAG	2,690.24	±473.80	—	2,748.94	±558.22	—	2,809.45	±510.34	—	2,649.53	±440.79	—
	iAG	2,610.56	±244.30	—	2,748.18	±262.08	—	2,837.39	±166.18	—	2,671.44	±481.00	—
Body weight (kg)	Ctrl	79.28	±12.83		78.27	±12.69		76.87	±12.72	†	79.59	±13.29	—
	cAG	71.36	±9.92	—	70.64	±9.92	—	69.74	±9.78	†	71.89	±10.02	—
	iAG	71.51	±4.62	—	70.88	±4.99	—	70.08	±5.43	†	72.18	±5.76	—

*significant difference from BDC‐3 (*p* < 0.05), ^†^significant difference from HDT3 (*p* < 0.05), ^a^symbolizes a significant difference from Ctrl (*p* < 0.05).

**FIGURE 6:**
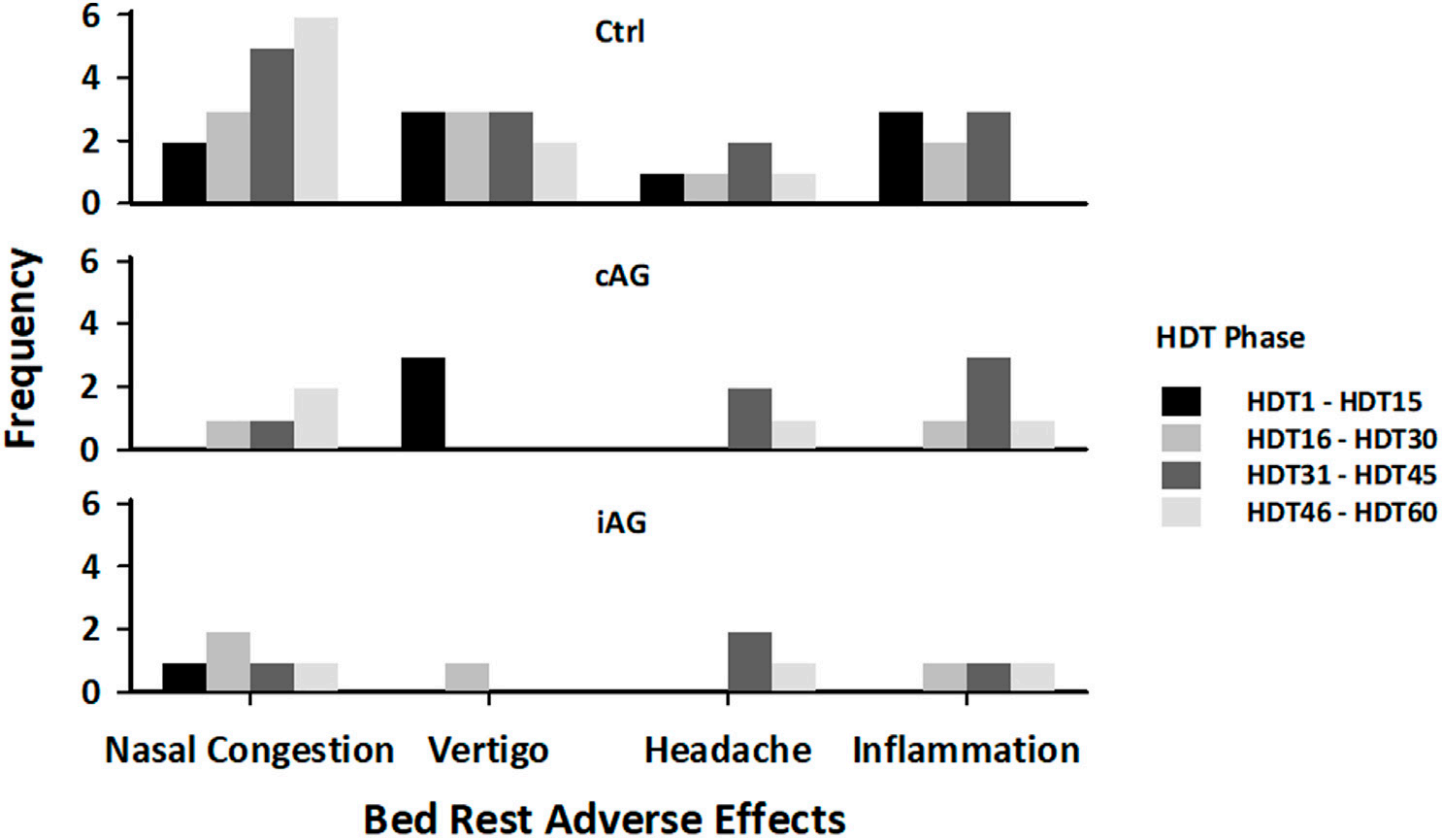
Frequency of adverse effects during the HDT phase for each subject group.

Bed rest related adverse reactions that required medical intervention included nasal congestion, vertigo, headache, and inflammation, and the frequency of these events are shown in Figure 6. These symptoms are shown because they occurred frequently (>10 incidents) during the HDT phase. Vertigo included dizziness and nausea; inflammation included infection of the ears, sinuses, eyelid, and upper respiratory airways. Obstipation (particularly at the beginning of HDT), gastro-esophageal reflux, and dry eyes occurred less frequently. Other bed rest related complaints included muscle pains in the neck, shoulders, back, and knees, and gastrointestinal tract problems.

Two additional countermeasure-related adverse reactions were persistent pain in the lower legs and feet (*n* = 1) and in the lower back (*n* = 1) during centrifugation. However, these adverse reactions did not result in premature cessation of the centrifugation sessions. The main complaint during the centrifugation sessions was pain at the calf muscle biopsy site.

Standard ocular safety measures during the HDT phase included bi-weekly assessments of intraocular pressure and near visual acuity. Weekly ocular monitoring during HDT was recommended by the ophthalmologist for 12 individuals because clinical suggestion of optic disc edema arose. Optic disc edema develops in astronauts during long-duration spaceflight, potentially due to headward fluid shift (Mader et al., 2011). Table 6 shows the results of optic disc edema evaluation on the first day of optic disc edema diagnosis, the demographic information of these individuals along with the HDT58, R+0, and R+1 evaluations of the optic disc edema using fundoscopy and graded according to the Frisen Scale (Frisen, 1982). Interestingly, no subject in the control group showed signs of an optic disc edema greater than grade 0.

**TABLE 6:** Results of optic disc edema evaluation at the end of the 60-days HDT phase. OD (*oculus dexter*): right eye; OS (*oculus sinister*): left eye; ODE Dx: optic disc edema diagnosis. Frisén grade 0 corresponds to an optic disc edema of up to 2/3 of the disc, grade 1 corresponds to an optic disc edema >2/3 of the disc.

ID	Group	Age (yr)	Sex	Height (cm)	Weight (kg)	OD	OS	1st day of ODE Dx
4	Ctrl	46	Female	183.3	79.55	grade 0	grade 0	HDT45
5	Ctrl	39	Male	182	87.22	grade 0	grade 0	HDT45
6	Ctrl	42	Male	179.5	75.69	grade 0	grade 0	R+0/1
9	Ctrl	30	Male	176.4	80.73	grade 0	grade 0	R+0/1
15	Ctrl	27	Male	171.2	72.68	grade 0	grade 0	HDT58
3	cAG	34	Female	174.5	75.41	grade 0	grade 0	HDT23
12	cAG	54	Male	174.4	77.16	grade 1	grade 1	HDT20
22	cAG	27	Male	175.3	73.11	grade 0	grade 0	HDT34
23	cAG	25	Male	173.2	71.27	grade 0	grade 0	HDT58
13	iAG	26	Female	166	71.43	no edema	grade 0	HDT31
19	iAG	29	Male	174.5	71.71	grade 0	no edema	HDT31
20	iAG	54	Male	188.7	78.47	grade 0	grade 0	R+0/1

### Artificial gravity countermeasure adherence and compliance

The adherence to the AG countermeasure in terms of attendance and exposure duration, as well as the ground reaction forces under the subjects’ feet, were recorded daily. Most participants performed static toe or heel raises during centrifugation, which did not generate any discernable ground reaction forces. In limited cases more dynamic strategies, such as shallow knee bends, were adopted to activate the skeletal muscle pump. The frequency at which the subjects engaged in such maneuvers to maintain tolerance during centrifugation vastly varied between the subjects and reflected individual differences in tolerability to the centrifugation (Kramer et al., 2020). Overall, no overt increase in muscle activation was observed over the course of the 60-days AG interventions.

Of the total 960 centrifugation sessions, only 12 sessions (1.25%) involving seven different individuals were interrupted (Frett et al., 2020). Of these 12 sessions, nine cAG sessions and one iAG session were aborted prematurely, and two sessions (1 cAG, one iAG) resumed after a short break, Reasons for discontinuing included 1) medical stop due to presyncopal signs (*n* = 8); 2) a high level of motion sickness including nausea/dizziness (*n* = 3); and 3) pain in bilateral lower legs (*n* = 1). The motion sickness susceptibility of these seven individuals ranged between 0 and 53.26th percentile of the population norm.

### Motion sickness susceptibility

A motion sickness susceptibility questionnaire (MSSQ-short) (Golding, 2006) was administered on BDC-10. The MSSQ-short captures the subject’s experience of malaise, nausea, and vomiting as it relates to certain types of motion (e.g., up-down translation, yaw rotation, etc.). The subjects retrospectively rated the frequency of sickness experienced in nine different modes of transportation from 0 (= never) to 3 (= frequently) during childhood (MSSQ-Child, <12 years of age) and adulthood (MSSQ-Adult, in the last 10 years). To aid in the interpretation of results, the total MSSQ raw scores (MSSQ = MSSQ-Child + MSSQ-Adult) were also evaluated as percentiles of the population norm provided by Golding (*N* = 257, age 26.0 ± 7.5 years) (Golding, 2006).

Table 7 lists the childhood, adulthood, and overall motion sickness susceptibility scores of the AGBRESA participants. No statistically significant difference was detected in the mean MSSQ-Child, MSSQ-Adult, and MSSQ score for the three experimental groups [*F* (4, 40) = 0.331, *p* < 0.856; Wilk’s *Λ* = 0.937, partial η^2^ = 0.032]. The participants’ motion sickness susceptibility before bed rest was within the population norm. In fact, the participants’ highest motion sickness susceptibility score ranked in the 72.06th percentile of the population norm.

**TABLE 7:** Motion sickness susceptibility scores of the AGBRESA participants at BDC-10 per experimental group as evaluated using the MSSQ questionnaires.

	MSSQ-child mean ± SD	MSSQ-adult mean ± SD	MSSQ mean ± SD	MSSQ percentile min - max
Ctrl	3.23 ± 2.81	1.54 ± 2.26	4.77 ± 3.85	5.06–45.20
cAG	3.82 ± 2.94	1.97 ± 1.55	5.79 ± 3.85	5.06–52.75
iAG	2.58 ± 3.77	2.09 ± 3.63	4.67 ± 7.04	0.00–72.06

### Artificial gravity countermeasure acceptability

As an evaluation of countermeasure acceptability, a modified Physical Activity Enjoyment Scale (Kendzierski and DeCarlo, 1991) was administered to evaluate the subject’s intrinsic motivation (e.g., pleasure, excitement, and interest), values that would influence their continuous engagement in the AG sessions. The AG countermeasure acceptability assessments were conducted weekly during the HDT phase. The 18-item questionnaire was tailored to assess the centrifugation experience in domains such as enjoyment of the AG sessions, AG effect on physical conditions, mood, vitality, and the subject’s desire to continue with the AG sessions. The subjects rated their experience on a 5-point Likert scale (1 = completely disagree; 5 = completely agree) and provided descriptions of the physical consequences of AG that focused on changes in typical bed rest-related symptoms such as headaches and/or pressure in the head, back pain, dizziness, and feeling unwell. Eight items were reverse scored so that higher scores represent greater levels of enjoyment, and the total score was converted to percentage of the theoretical maximum 90 points.

**FIGURE 7:**
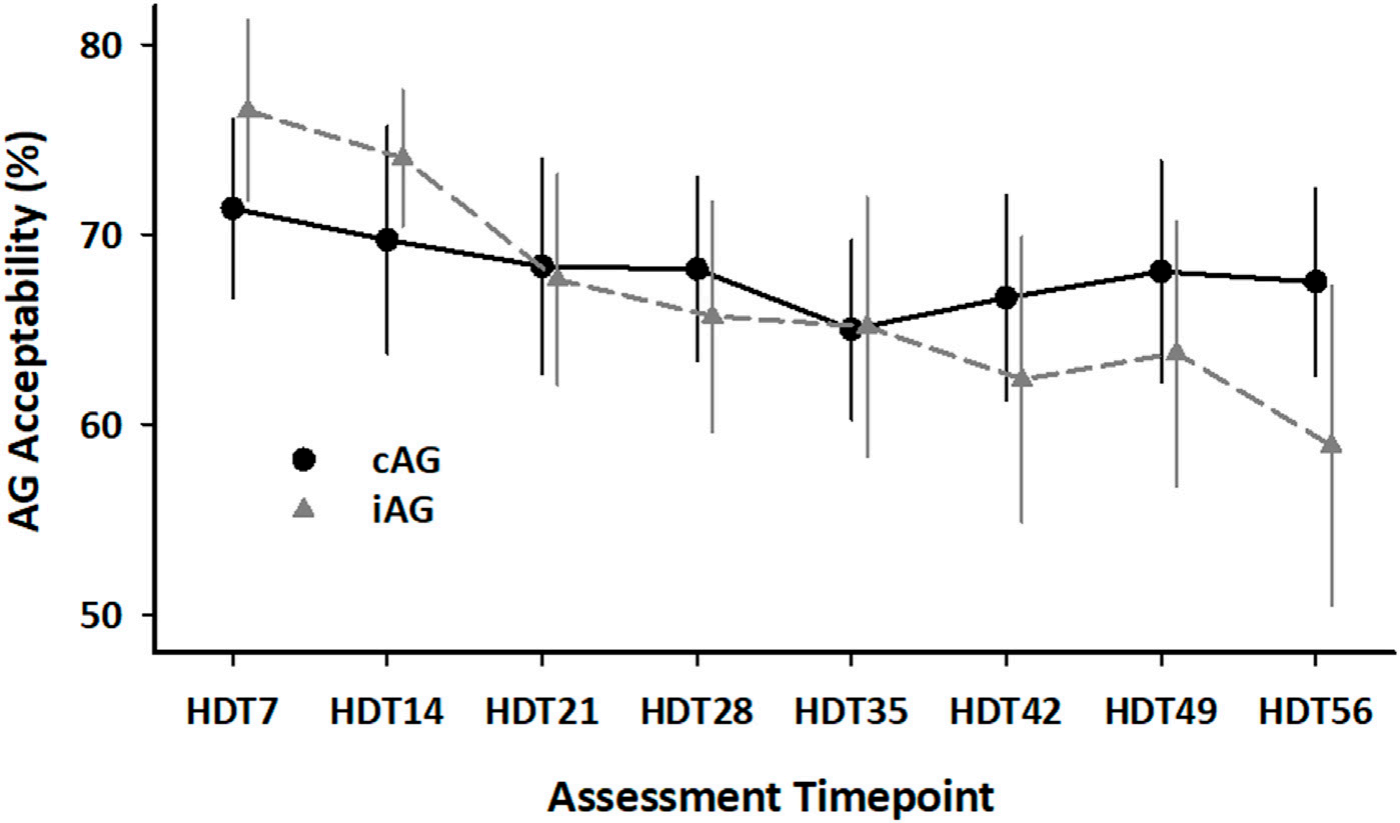
Average acceptability of the AG countermeasure measured in weekly intervals for the cAG and iAG subject groups (8 subjects per group). Mean ± SD.

Figure 7 shows the average percentage enjoyment of the centrifugation sessions as an evaluation of countermeasure acceptability measured in weekly intervals. No statistically significant differences were detected between the three subject groups on the individual assessment timepoints, but the iAG group showed a significant decline in the enjoyment ratings with the repetition of the runs, whereas the cAG group did not (group x time: *β* = 0.251, *p* = 0.01; group: *β* = −5.095, *p* = 0.47; time: *β* = −0.325, *p* < 0.01).

Reported positive effects of centrifugation included stimulation of the legs, relieved back pain, improved digestion, reduced bloating, and increased energy. The reported negative effects of centrifugation largely overlapped with those captured during the clinical evaluation above. Additional reports of discomfort included feeling of warmth, unwellness (e.g., dizziness, nausea, increased heart rate), fatigue, and pressure in the head. Most of these negative comments were clustered towards the later stage of the HDT phase (>HDT28).

### Neurovestibular Symptoms

Subjective ratings of the severity of neurovestibular symptoms experienced upon re-ambulation were collected at the end of the day on R+0 and R+3. The subjects were presented with the identical questionnaire used by NASA to review the list and severity of neurovestibular symptoms experienced by crewmembers upon returning from space (Richards et al., 2002). The questionnaire included a list of 14 potential symptoms that the subjects were asked to rate on a scale from 0 to three according to their severity (0 = none; 1 = mild, symptom awareness; 2 = moderate, symptom present, no performance impact; 3 = severe, symptom present and interferes with performance). Symptoms were off balance, feel abnormally heavy, problems when making rapid head movements, clumsiness, dizziness, difficulty turning corners, motion illusions, sweating, nausea, stomach awareness, malaise/sluggishness, disorientation, vomiting, and dry heaves. A composite Re-adaptation Symptom Severity (RSS) score was tabulated as the sum of the scores for the 14 items.

**FIGURE 8:**
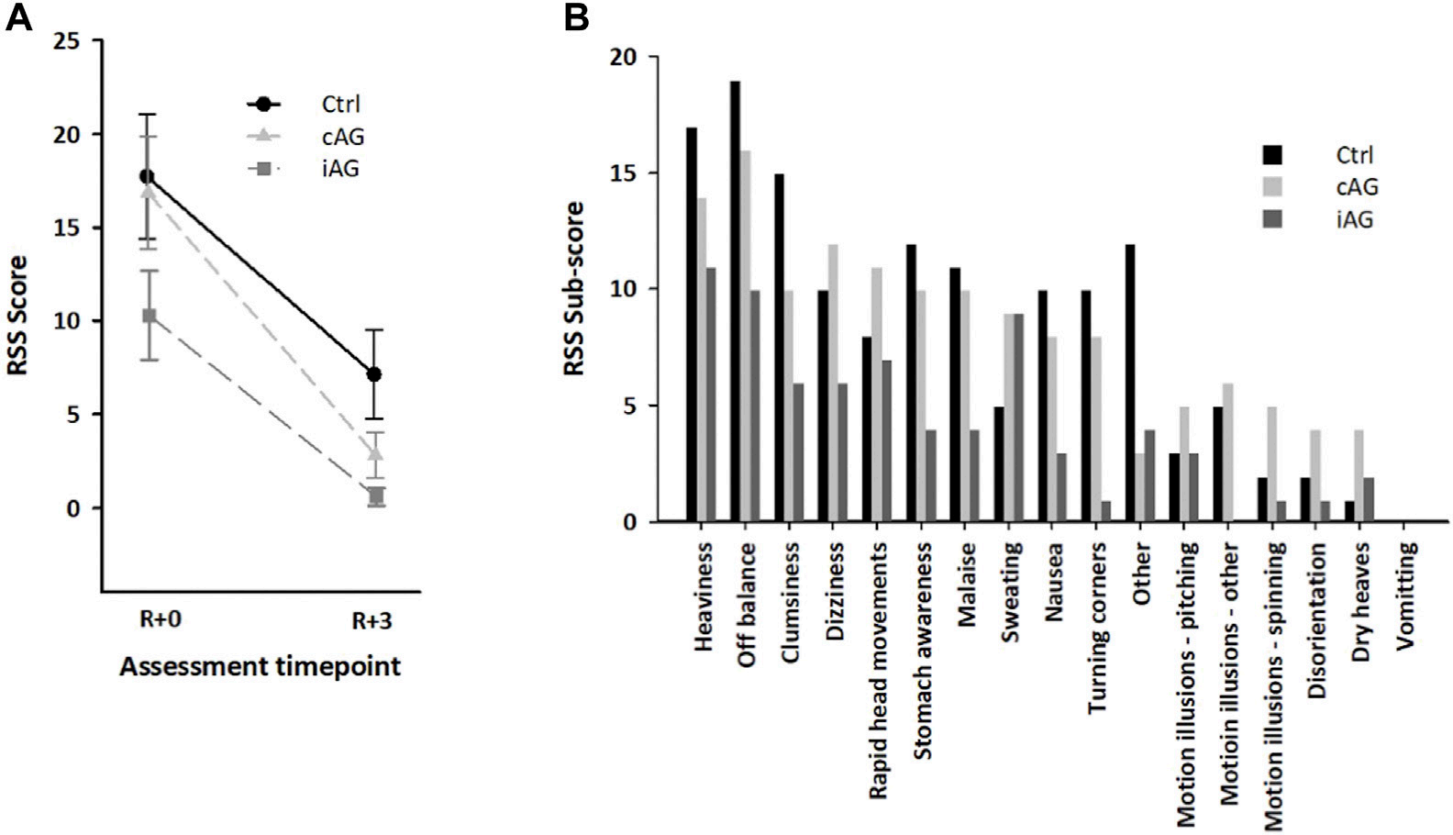
**(A)**. Mean ± SD of the composite Readaptation Symptom Severity (RSS) scores in the three subject groups on R+0 and R+3. **(B)**. RSS sub-scores in the three subject groups for neurovestibular and other symptoms on R+0.

The rate of change in the RSS scores between R+0 and R+3 was not significantly different for the three subject groups (group x time: *F* = 1.306, *p* = 0.292; group: *F* = 4.049, *p* = 0.026; time: *F* = 51.561, *p* < 0.001) (Figure 8A). All but one participant (iAG, m) reported neurovestibular effects on R+0. A significant difference in the RSS scores among the three experimental groups was only detected at R+3 [*F* (2, 19) = 4.180, *p* < 0.031, partial η^2^ = 0.306]. A pair-wise *post hoc* test revealed that the RSS score was significantly higher in the Ctrl group than in the iAG group (Bonferroni corrected *p* = 0.03) on R+3.

Figure 8B shows the RSS scores collected for each neurovestibular symptom on R+0 for each of the three subject groups. Other symptoms were also reported, such as muscle and joint pain in the back and lower extremities, swollen ankles, difficulty concentrating, and short-term memory lapse.

## Discussion

### Lessons learned from artificial gravity bed rest study with the european space agency

AGBRESA was the most complex integrated bed rest study conducted in a joint effort by NASA, ESA, and the DLR to date. Implementation of strict HDT, diet, test timing, and test constraints were well controlled throughout the study. This standardization of the study platform enables comparison of the countermeasure efficacy among bed rest studies. A good example of this capability is the following. In 2017, DLR hosted the Vision Impairment and Intracranial Pressure and Psychological:envihab Research (VaPER) study, which assessed the effects of 30-days HDT bed rest combined with elevated ambient CO_2_ levels. VaPER was the first spaceflight-analog study in which nearly half of the participants developed ocular changes akin to the Spaceflight Associated Neuro-ocular Syndrome (SANS) (Laurie et al., 2019). SANS is observed in approximately one third of astronauts after long-duration missions (Lee et al., 2016). However, the absence of head pillows during bed rest was also introduced during the VaPER study, which could have potentially exacerbated cephalad fluid shift. Consequently, the effects of elevated CO_2_ exposure on SANS during the VaPER study were inconclusive (Lawley et al., 2017; Clément et al., 2022). Because no pillows were used during the AGBRESA study, the control subjects who were not exposed to AG can serve as a reference group for the VaPER study, and disentangle the effect of elevated ambient CO_2_ from effects due to strict HDT bed rest. No subject in the AGBRESA control group showed signs of SANS, suggesting that the elevated CO_2_ exposure could have be responsible for the development of ocular changes in some of the VaPER subjects (Laurie et al., 2021).

The 60-days bed rest phase of AGBRESA was intended to extend and complement knowledge gained from the previous four- to 21-days AG bed rest studies (Yajima et al., 1994; Iwasaki et al., 1998; Iwase, 2005; Linnarsson et al., 2015). Indeed, a longer bed rest phase enables the efficacy of AG to be evaluated on bone, muscle structure and strength, and sensorimotor deconditioning, which are more pronounced during longer durations of HDT bed rest. Additionally, in AGBRESA, the participants rated the acceptability of the various AG interventions. These responses had not been assessed in prior AG bed rest studies but are essential for the success of exploration-type space missions. Moreover, in contrast to the previous AG bed rest studies, which were conducted exclusively in men, AGBRESA was designed to have equal sex representation.

AGBRESA demonstrated that strict HDT bedrest (i.e., no pillow usage) combined with daily 30-min supine centrifugation was reasonably well accepted by the participants for 60 consecutive days. Overall, the iAG protocol had a higher completion rate and had comparable or more physical benefits than the cAG protocol. However, subjects experienced less time in the HDT position in the iAG protocol than in the cAG protocols (iAG subjects were kept in the horizontal position between consecutive centrifugation runs), which could have contributed the increased physical benefits in the iAG protocol. Nevertheless, the subjective acceptability of the iAG protocol significantly decreased over the course of 60 days.

Critical parameters for the trade-off between AG acceptability and effectiveness are duration of AG exposure and AG intensity (Clément, 2017). The AGBRESA design included daily 30-min centrifugation that induced 1 Gz at the CoM, which was well tolerated by the participants. A factor in this acceptability was presumably the engagement of prophylactic skeletal muscle pumps during the AG intervention to prevent presyncope. Additionally, familiarization sessions conducted during both the recruitment process and the BDC phase may have accounted for the high completion rate of the AG countermeasure. By contrast, invasive experimental procedures (e.g., muscle biopsy, microdialysis) performed on the lower extremities often led to pain during centrifugation, compromising the subject’s muscle pump performance against orthostatic intolerance. Thus, in future AG protocols, careful considerations with regards to pain management and coaching of muscle pumps should be made when invasive experiments on the lower extremities are combined with supine centrifugation.

AGBRESA experiments were integrated to avoid confounding factors and to accommodate constraints such as food intake, activity, and the time of the day of each measure. The R+0 schedule minimized redundant examinations and increased test validity while reducing risk of injuries. Still, the test schedule on R+0 was extremely packed and not all tests were completed by all subjects, mainly due to the subjects’ limited physical capacity. Performance on tests administered later in the day was complicated by fatigue and muscular pain associated with re-ambulation and physical exertion following prolonged restricted mobility. This resulted in several injuries (e.g., knee and ankle joint injury from landing during jumping), which in turn affected the following R+ phase testing, reconditioning, and eventually recovery in these subjects. To support test performance on R+0, sufficient rest time must be implemented between experiments to allow the subjects to recuperate from physical and psychological exhaustion. Sharing data between studies would reduce redundant testing of near-identical measures with unique setups on R+0. In addition, experiments requiring high acceleration loads on the lower body, such as treadmill running and drop jump tests, should be avoided on the day of re-ambulation. Moreover, experiments that are known to have a steep learning curve should be shortened with minimal repetitions.

Maintaining the physical and psychological health of bed rest subjects is imperative for subject safety and for successful completion of the study. We recommend conducting consistent safety tests and long-term follow-up measures such as those implemented in AGBRESA to keep the subjects safe and to monitor their recovery. When the head position is strictly controlled, such as during strict HDT, subjects experience more incidences of vertigo while being transported than subjects of previous bed rest studies. If an additional baseline measurement of neurovestibular symptoms is included, this would allow pre-to post-bed rest change in susceptibility to be assessed, expanding the utility of the measure beyond just evaluation the re-adaptation syndrome. Some participants experienced adverse reactions such as persistent severe vertigo or difficulties linked to food consumption early in the HDT phase that were are particularly challenging for them. Effective preventive measures, such as maneuvers for alleviating positional vertigo with minimal disruption of the HDT effect, and new strategies to manage difficulties with food and fluid intake while maintaining comparability between studies, are needed. Finally, bed rest participants are confined to bed, with limited autonomy over their schedule, isolated from their home and community, and deprived of privacy and customary amenities (Harrison, 2005). It is therefore important to conduct sensitivity training sessions with staff and investigators to inform them of the frustrations the participants may experience, and to promote research aimed at understanding the impact of strict HDT bed rest on psychology.

### Study limitations and future directions

Due to the high operational cost and the challenging nature of complex bed rest studies, the number of participants is kept to the bare minimum required for addressing primary outcomes. Consequently, the low sample size, in this case 8 subjects per experimental group, may have limited the statistical power to detect relevant effects. We anticipate that data pooling across several bed rest studies, which is possible if standard conditions and procedures are implemented, would alleviate this issue to a certain degree. Further, despite our concentrated effort to engage more women, we could not reach the target of recruiting equal numbers of men and women in the AGBRESA study. As a result, the data suffered from imbalanced sex distribution among the experimental groups, limiting the opportunity to adequately assess potential sex differences in AG efficacy.

The AGBRESA AG protocol allowed the subjects to contract their muscles as an anti-gravity straining maneuver. Muscle contraction may have limited the efficacy of the AG countermeasure for some cardiovascular parameters and cephalad fluid shifts implicated in the development of SANS. Muscle pumping actively pushes the venous blood to the heart, increasing preload and stroke volume (Sparks and Rooke, 1987). This effective reverse of blood flow may have partially negated the targeted caudal fluid shift induced by centrifugation. Moreover, the high central blood volume maintained through muscle pumping may have interrupted the protective process necessary to halt plasma volume loss, which was expected to be triggered by a temporary decrease in central blood volume with centrifugation. Passive centrifugation (i.e., without activation muscle pumping) may potentially be more effective in counteracting adverse effects of headward fluid shifts and loss of plasma volume (Goswami et al., 2015). However, passive centrifugation could result in more frequent AG interruptions, which would make it difficult to compare the effects of cAG and iAG. Future studies need to take into consideration this trade-off when establishing the AG protocol to evaluate AG efficacy.

## Data Availability

The raw data supporting the conclusion of this article will be made available by the authors, without undue reservation.
