# Supplementary material for: Assessing the effects of artificial gravity in an analog of long-duration spaceflight: The protocol and implementation of the AGBRESA bed rest study
Source: Front Physiol. 2022 Sep 8;13:976926. doi: 10.3389/fphys.2022.976926 (PMC9492851; doi:10.3389/fphys.2022.976926)
Supplement: Supplementary file 1 [file Table1.DOCX]

**Supplementary material**

NASA, ESA, and DLR selected research projects included in AGBRESA as part of the science complement. These investigations, which address different specific aims, were integrated into the standardized core study structure on a non-interference basis, taking into account the restrictions and assessment time point specified and proposed by the principal investigators. The results of these studies will be published in separate articles.

| **No.** | **Title** | **Measures** |
| --- | --- | --- |
| 1 | Studying the Physiological and Anatomical Cerebral Effects of Centrifugation and Head Down Tilt Bed Rest | Non-invasive brain and ocular physiological monitoring systems to assess the cerebral, ocular, and vestibular systems |
| 2 | Hyper-Campus – Effects of Artificial Gravity on Structural and Functional Plasticity During Head-Down Tilt Bed Rest | Spatial learning and memory during cognitive tests, various neuroimaging scans. blood and saliva samples. |
| 3 | Does Intermittent or Continuous Artificial Gravity Counteract Long Duration Bed Rest Induced Neurocognitive Declines? | Brain imaging activities, behavioral tests, neuromuscular tests, neurovestibular response tests, and neuropsychological assessments. |
| 5 | The effects of a Novel Exercise Device for the Rehabilitation of Lumbo-pelvic Musculoskeletal Adaptations, Postural Control, and Low Back Pain Following Long-Term Bed Rest | Muscle size, muscle function, disc height, balance and pain via MRI scans, ultrasound scans, EMG and questionnaires. |
| 6 | Physical Performance and Biological Age | Muscle performance assessments with surface EMG, ground reaction force data, blood biomarkers. |
| 7 | Effectiveness of Artificial Gravity to Maintain Muscle Strength and Neuromuscular Interaction during 60 days of Bedrest | Muscle functional units and muscle wasting as evaluated via muscle biopsies, blood draws and electrical stimulation of muscles. |
| 8 | Effectiveness of Artificial Gravity Against Musculoskeletal Deterioration and Pain during Bed Rest Compared to Previously Yested Countermeasures and Control | DXA, body composition, pQCT, XTremeCT, QCT, MRI, blood draws, microdialysis, balance and exercise assessments, and questionnaires. |
| 9 | Role of Interleukin-6-induced Hepcidin Overexpression in Altered Iron Metabolism Induced by Prolonged Bed Rest: Preventive Effect of Artificial Gravity? | Iron metabolism via blood draws. |
| 10 | Cardiac Contractility and Autonomic Adaptation in Simulated Microgravity with Artificial Gravity | Heart MRIs, kineticardiography, heart kinetics self-assessments, and echocardiography. |
| 11 | Effect of Artificial Gravity Regimens on Neurocognitive Performance During Head Down Tilt Bedrest | Questionnaires, visual and auditory cognition tasks and neurocognitive measurements (EEG and fNIRS). |
| 12 | Artificial Gravity Loading Assessment | Ground reaction forces, medical monitoring, subjective rating of the countermeasure ascertained via questionnaires. |
| 13 | Wound Healing Assessment | Muscle biopsy wounds from the vastus lateralis and soleus muscles, wound dimensions, color and thermography imaging, subjective reports (e.g. pain). |
| 14 | Orthostatic Tolerance | Aortic vessel wall properties, central blood pressure, and cardiac function and structure. |
| 15 | Plasma Volume | Plasma and blood volume |
| 16 | DNA Repair Capacity During Bed Rest | Inter-individual variability of DNA repair capacity of human lymphocytes. |
| 17 | Head Infections During Head Down Tilt Bed Rest | Infections of the head, as otitis externa, sinusitis or conjunctivitis |
| 18 | Effects of Bed Rest and Artificial Gravity on Natriuretic Peptides and Metabolic Changes | Level of natriuretic peptides (NP) secretion with blood draws and metabolic changes. |
| 19 | Self-Sufficient 6df Learning Program | Docking performance level by using a Russian regular docking training system. |
| 20 | Assessment of Autonomic Response Pattern for individualized Analysis of Physiological Centrifuge Data | Inter-individual comparability of a Psychophysiological Arousal Value (PAV) |
| 21 | Sarcolab and Bed Rest | Ankle and a knee actimetry, MRIs to assess muscle volume, soleus biopsy |
| 22 | Predictive Validity of the Subject Selection Process and the Role of Mood and Personality | Interviews and questionnaires |
| 23 | Sleep and Sleepiness | Repeated polysomnography and wake EEG |
| 24 | Eye Examinations: Visual Field Testing and objective refraction | Ocular measurements |
